# Supplementary figures and images for: Activation loop phosphorylation and cGMP saturation of PKG regulate egress of malaria parasites
Source: PLoS Pathog. 2024 Jun 27;20(6):e1012360. doi: 10.1371/journal.ppat.1012360 (PMC11236177; doi:10.1371/journal.ppat.1012360)

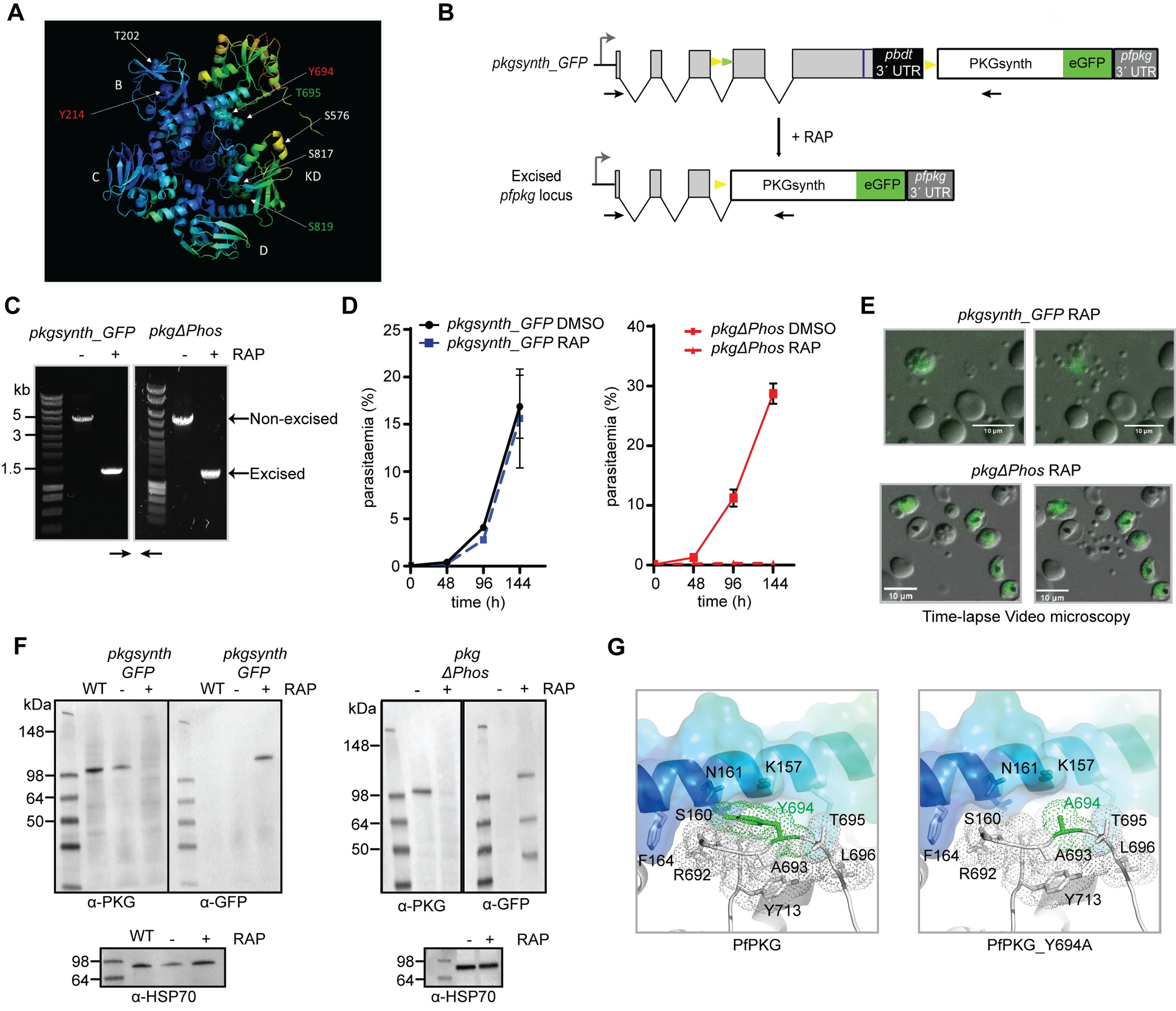

Supplement: S1 Fig — (A) Cartoon representation of the P. falciparum PKG x-ray crystal structure (PDB ID: 5DYK) in its apo form where predicted phosphosites are indicated and shown as sticks within colour matching spheres (B) Schematic representation of the approach used to create line pkgsynth_GFP. Blue line indicates the position targeted by the gRNA. RAP-induced DiCre activity switches expression from wt PKG to a gene replacement with a partially synthetic pfpkg gene fused to eGFP. Black arrows; oligonucleotides used for excision PCR. (C) Excision PCR showing generation of products after activation of DiCre upon RAP treatment in line pkgsynth_GFP and pkgΔPhos. Expected sizes of the amplicons corresponding to non-excised and excised fragments are 5kb and 1.4 kb respectively. (D) Growth curves showing replication of DMSO-treated (control) or RAP-treated pkgsynth_GFP and pkgΔPhos lines. Mean values are shown. Error bars: ± SD (n = 2). (E) Representative still images of a 30 min time-lapse videoof mock-treated (grey) or RAP-treated (green) parasites of lines pkgsynth_GFP and pkgΔPhos. pkgsynth_GFP RAP-treated schizonts underwent egress (upper panel), whilst pkgΔPhos RAP-treated parasites did not (lower panel). Scale bar, 10 μΜ. (F) (Left panel) Western blot showing expression of PKG (expected MW: 98 kDa) in DMSO-treated pkgsynth_GFP schizonts relevant to the parental line (WT). Upon RAP-treatment there is appearance of a signal corresponding to the PKG_GFP fusion (expected molecular weight: 125 kDa). (Right panel) Representative Western blot of pkgΔPhos schizonts after DMSO or RAP treatment. Note the multiple bands appearing at the RAP-treated sample. Cytoplasmic HSP-70 was used as a loading control. (G) Cartoon representation of PfPKG (PDB: 5DYK) with Y694 in green, the CNB-A to CNB-B connecting helix in a turquoise-blue gradient and the PfPKG kinase activation loop residues in white. (Left panel) The wild type PKG Y694 is engaged in stabilizing interactions with the helix residues K157 [file ppat.1012360.s001.tif]

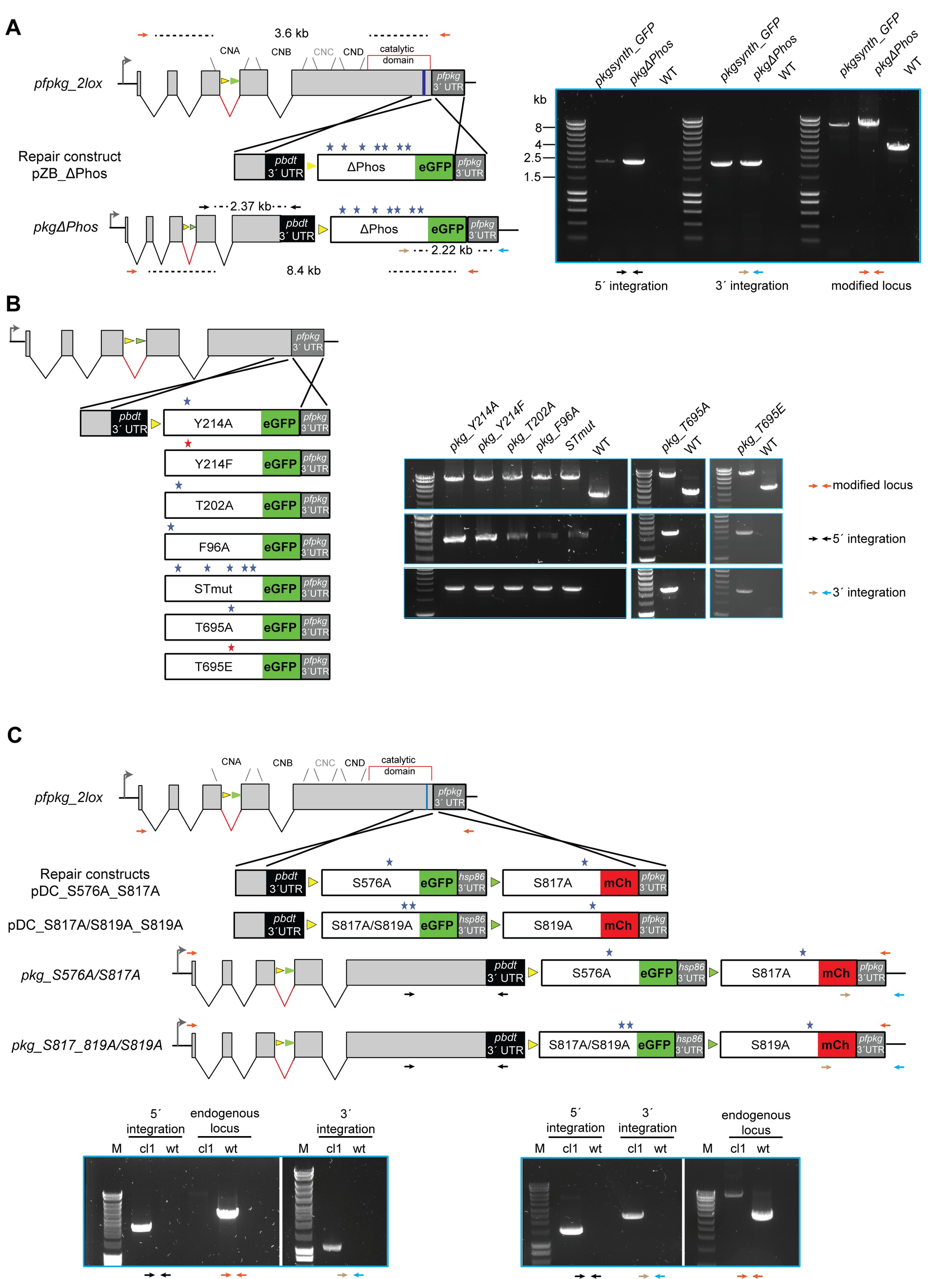

Supplement: S2 Fig — (A-B) Modification strategies and genotyping data for generation of the parasite lines pkgΔPhos, pkgY214A, pkgY214F, pkgT202A, pkgF96A, pkgSTmut, pkgT695A and pkgT695E respectively. Blue line indicates the position targeted by the gRNA. Relative positions of mutations are depicted by coloured stars. Positions of oligonucleotides used for genotyping by diagnostic PCR are indicated (coloured arrows), and agarose gel electrophoresis of corresponding PCR products are shown. Positions of lox sites are indicated with coloured arrowheads (yellow, loxN; green, lox2272). (C) Modification strategy for lines pkgS576A_S817A and pkgS817A/S819A_S819A. Blue line indicates the position targeted by the gRNA. Relative positions of mutations are depicted by coloured stars. Positions of oligonucleotides used for genotyping by diagnostic PCR are indicated (coloured arrows), and agarose gel electrophoresis of corresponding PCR products are shown. Positions of lox sites are indicated with coloured arrowheads (yellow, loxN; green, lox2272). (TIF) [file ppat.1012360.s002.tif]

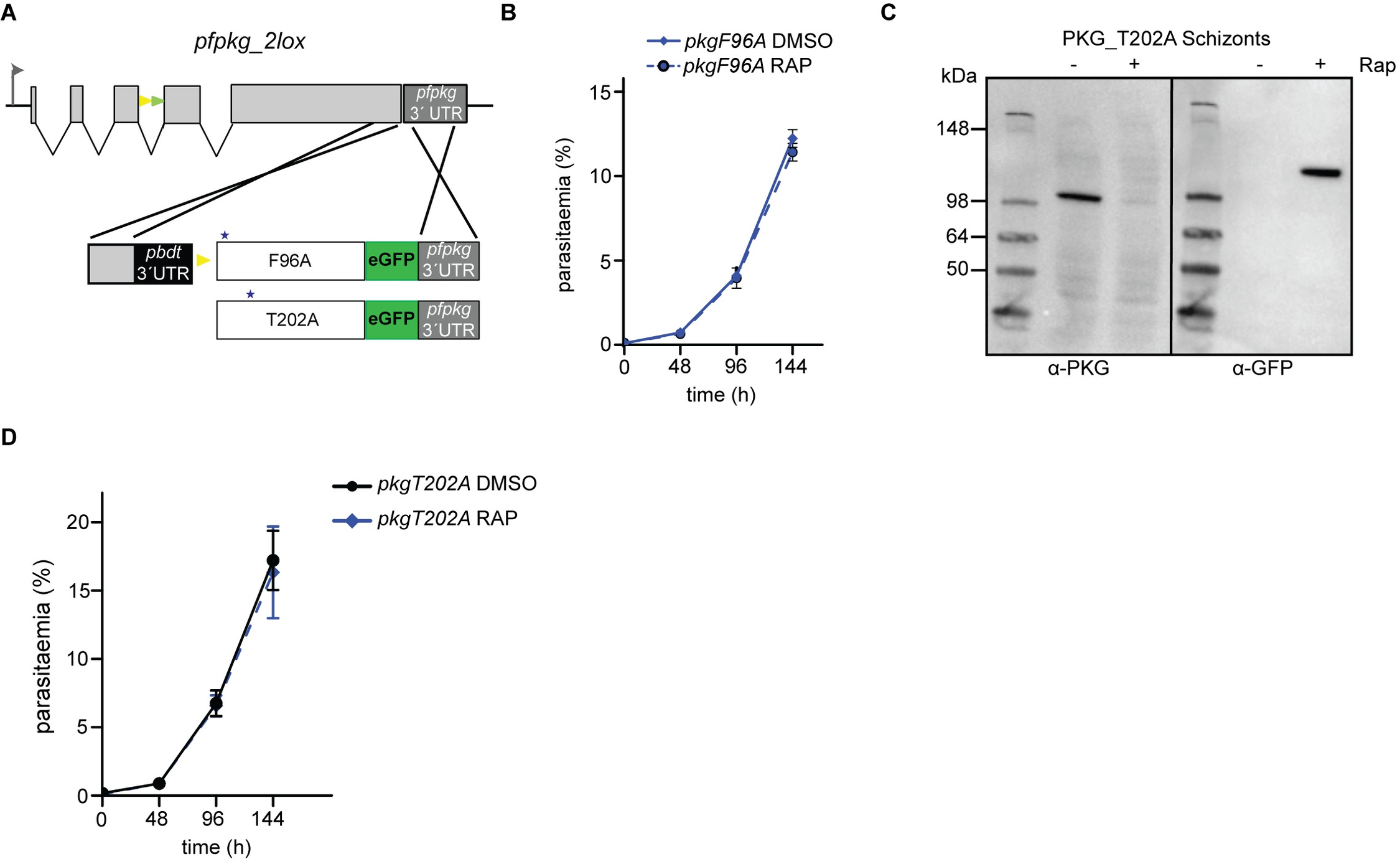

Supplement: S3 Fig — (A) Schematic representation of the approach used to create lines pkgF96A and pkgT202A. Relative positions of the mutated amino acids are denoted by blue stars. (B) Growth curve showing replication of DMSO-treated (control) or RAP-treated pkgF96A. Mean values are shown. Error bars: ± SD (n = 3). (C) Western blot showing expression of endogenous PKG in DMSO-treated pkgT202A schizonts and of the mutated PKG (T202A) fused to GFP upon RAP-treatment. (D) Growth curve showing replication of DMSO-treated (control) or RAP-treated pkgT202A. Mean values are shown. Error bars: ± SD (n = 3). (TIF) [file ppat.1012360.s003.tif]

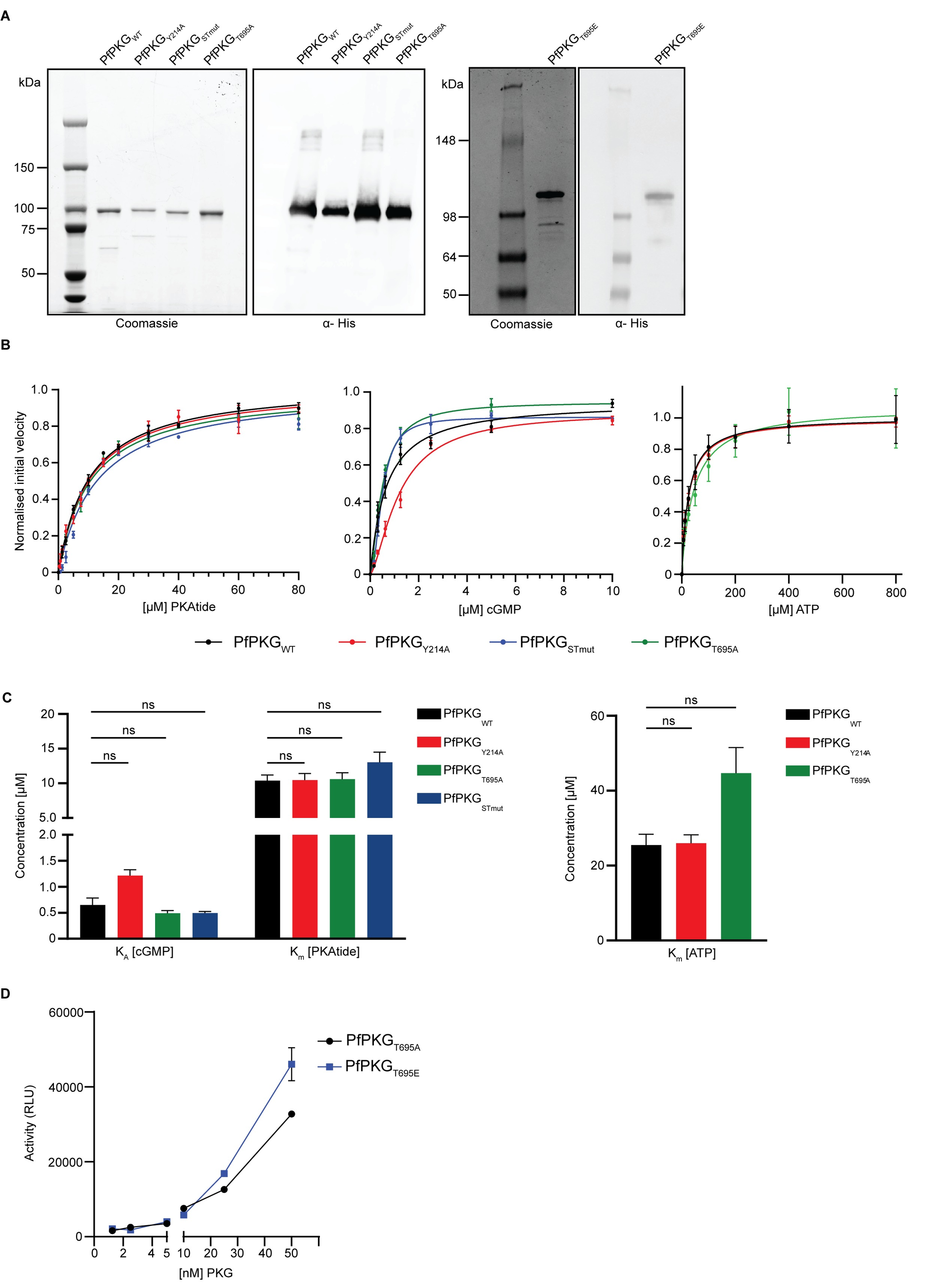

Supplement: S4 Fig — (A) Coomassie stained gels and western blot analysis of the 5 different recombinant PfPKGs used in this study. (B) Determination of PKAtide Km, cGMP KA and ATP Km for PfPKGWT, PfPKGY214A, PfPKGSTmut, PfPKGT695A. (C) Summary of kinetic parameters, shown are the means ± SEM and Two-way ANOVA. (D) PfPKGT695A and PfPKGT695E activity assay showing phosphorylation of PKAtide. (TIF) [file ppat.1012360.s004.tif]

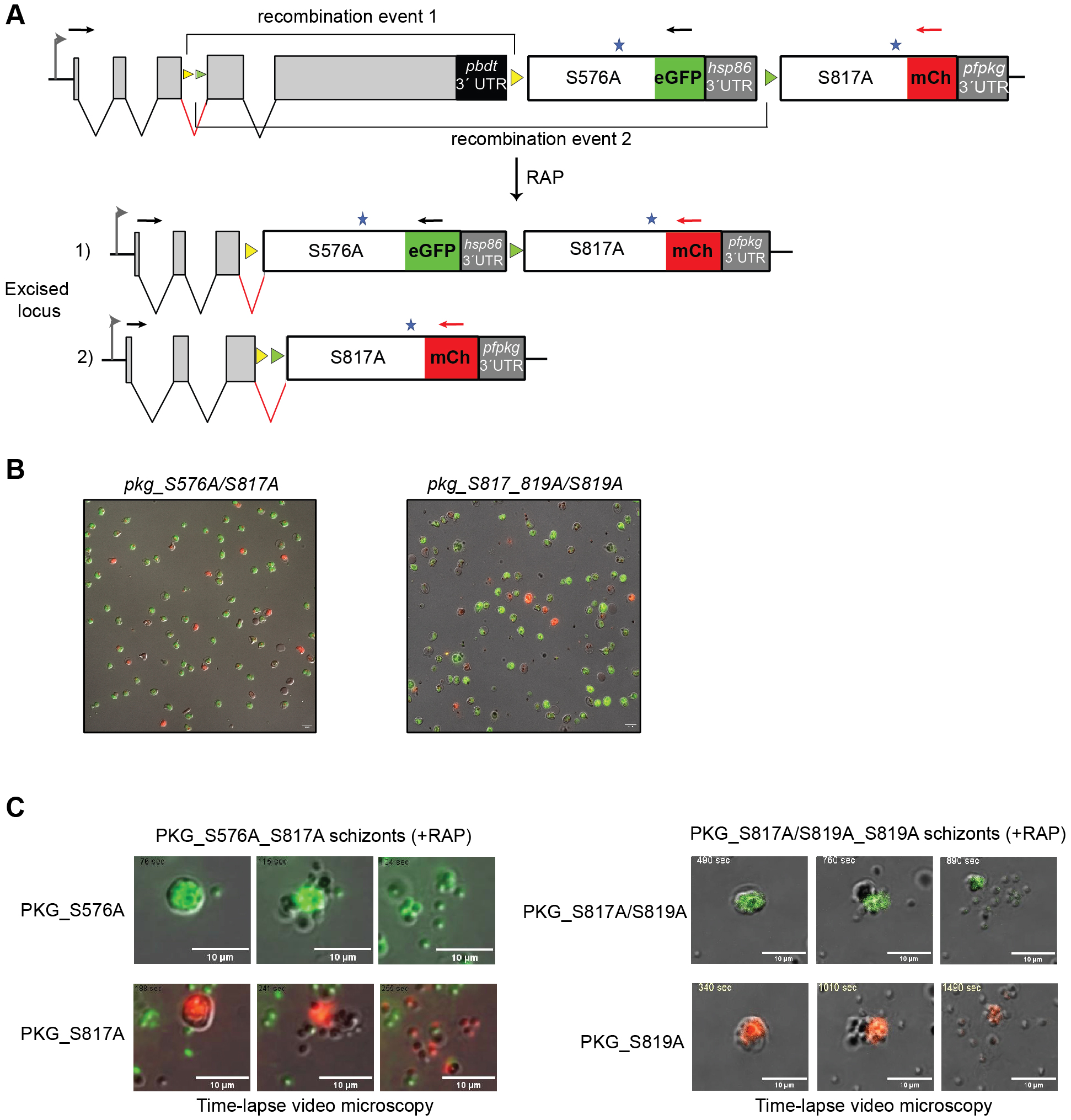

Supplement: S5 Fig — (A) Schematic of line pkgS576A_S817A. RAP-induced DiCre activity switches expression from wt PKG to either a gene replacement with a S576A mutant fused to eGFP (recombination event 1; PKG_S576A) or to expression of a S817A mutant fused to mCherry (recombination event 2; PKG_S817A). Similar allelic replacement strategy applies to line pkgS817A/S819A_S819A. Black and red arrows; oligonucleotides used for identification of both events by diagnostic PCR. (B) Representative images from DIC/fluorescence microscopic examination of RAP-treated pkgS576A_S817A and RAP-treated pkgS817A/S819A_S819A parasites (end of cycle 0), showing both GFP-and mCherry-positive schizonts. Scale bar, 10 μm. (C) Stills from time-lapse DIC/fluorescence microscopy of isolated, RAP-treated pkgS576A_S817A and pkgS817A/S819A_S819A schizonts, showing that all four mutants can undergo egress. Scale bars, 10μm. (TIF) [file ppat.1012360.s005.tif]

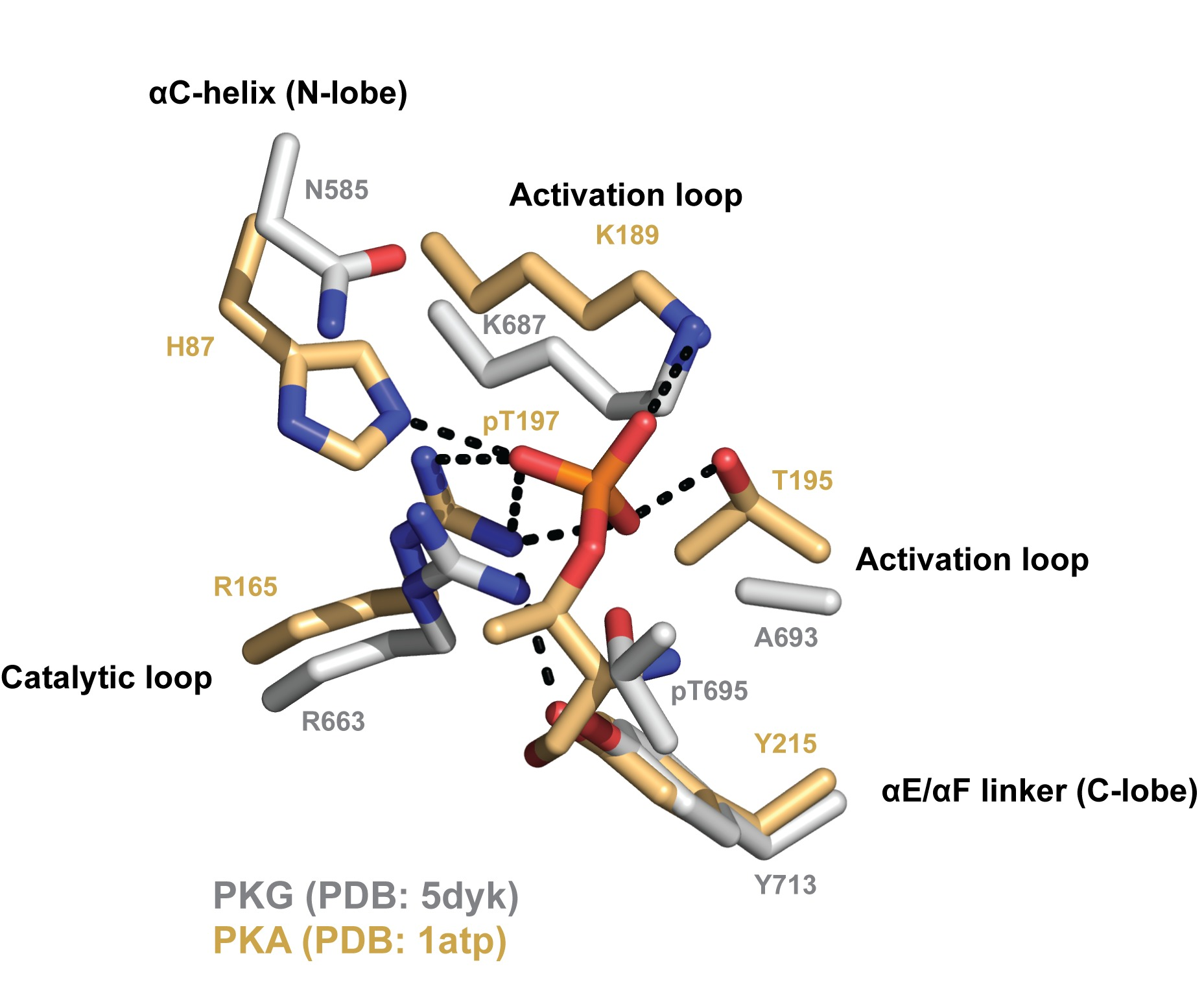

Supplement: S6 Fig — The molecular structures of PKG (light grey sticks coloured by elements, PDB: 5dyk) and PKA (mustard sticks coloured by elements, PDB: 1atp) were superimposed (rmsd: 1.3Å). The structurally equivalent residue side chains from the phosphorylation site are depicted here and labelled. Hydrogen bonds in the phosphorylated PKA are seen as black dashed lines with the phosphate group in orange. Structurally stabilizing PKA residues H87 (in the C-helix) and T195 (in the Activation loop) are not conserved in PKG (N585 and A693 respectively). (TIF) [file ppat.1012360.s006.tif]

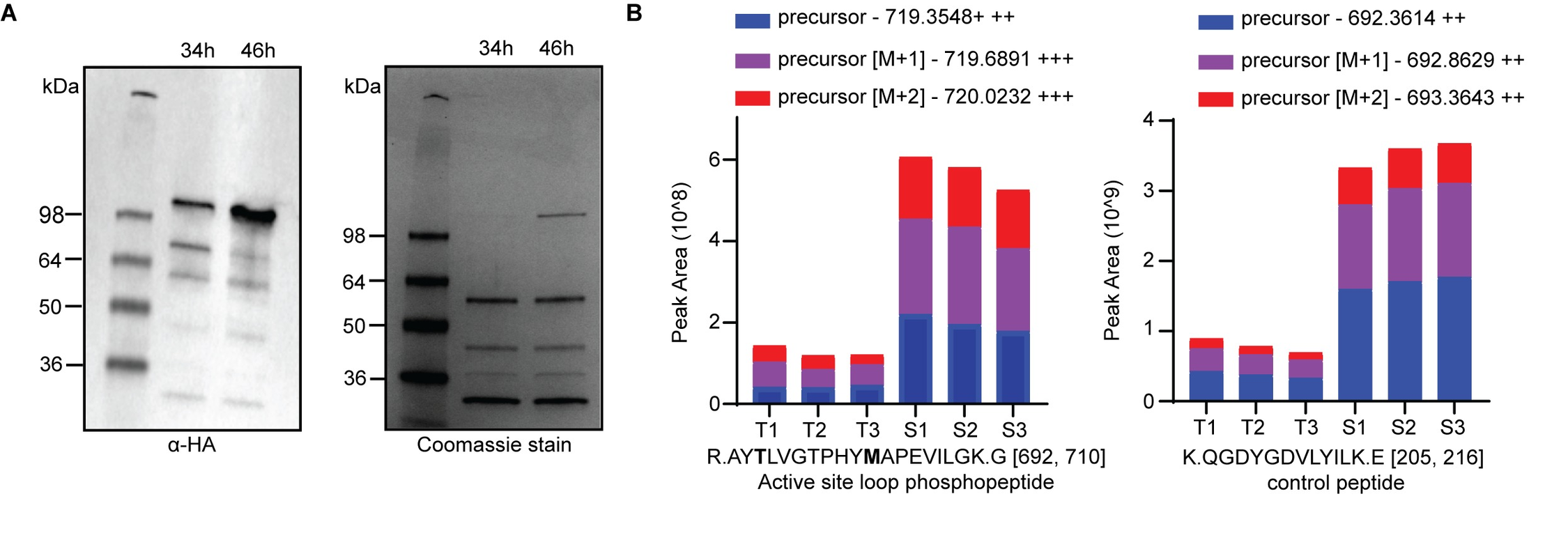

Supplement: S7 Fig — (A) Western blot and Coomassie stained gel after PKG pulldown showing presence of the protein in both trophozoites (34h) and schizonts (46h). (B) Peak areas of precursor ion chromatograms are extracted after MS1 filtering for peaks picked based on MS2 peptide identification in three technical replicates at both trophozoite (T1-T3) and schizont samples (S1_S3). Peaks for the phosphorylated T695 peptide were found in all experiments (Left panel) showing similar ratio between trophozoite and schizont samples as the control peptide (Right panel). (TIF) [file ppat.1012360.s007.tif]

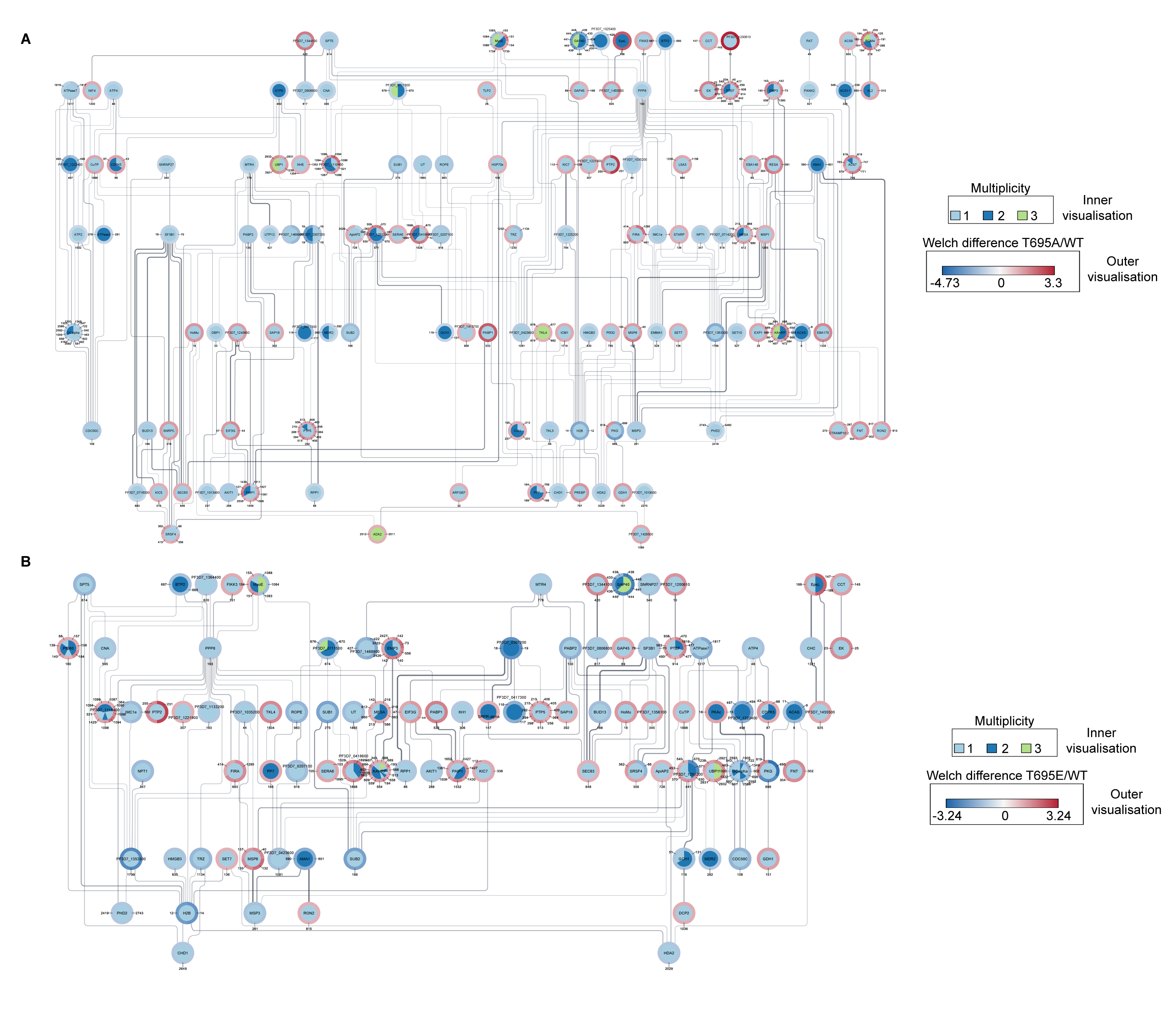

Supplement: S8 Fig — (A) STRING network (y Files Hierarchical layout) visualizing functional interactions (edges) between proteins (nodes) significantly dephosphorylated in the T695A mutant over WT. Default STRING clustering confidence score cutoff of 0.4 was used to determine whether two nodes were functionally related. Single proteins are excluded from the schematic. Significantly deregulated phosphosites are depicted on each protein and were coloured according to the Welch difference (outer visualisation) and the multiplicity (number of phosphorylation events in the peptide–inner visualisation). Names or PlasmoDB IDs are displayed on each protein. (B) STRING network (y Files Hierarchical layout) visualizing functional interactions (edges) between proteins (nodes) significantly dephosphorylated in the T695E mutant over WT. Same clustering parameters and visualisation were used as in (A). (TIF) [file ppat.1012360.s008.tif]

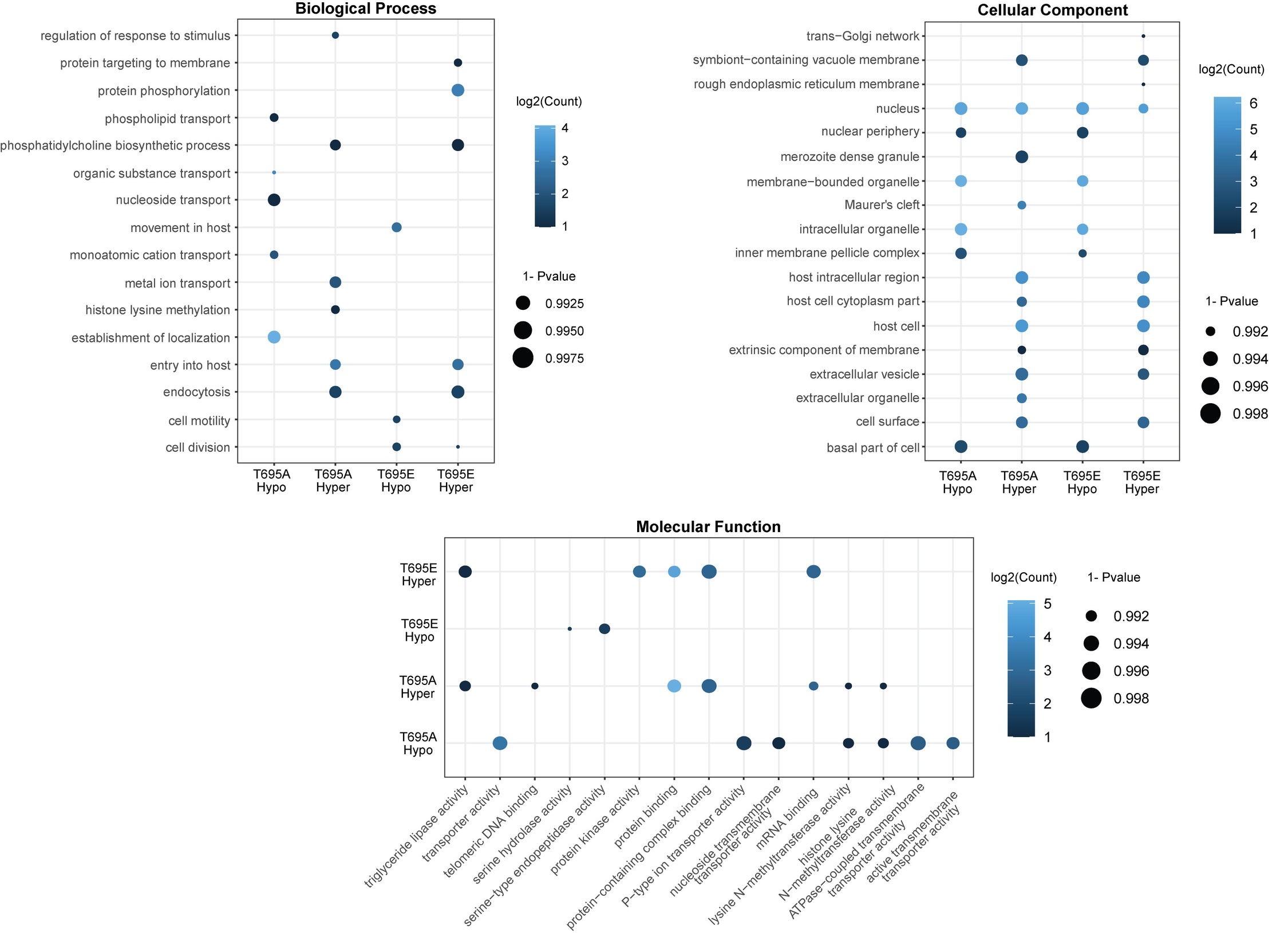

Supplement: S9 Fig — GO terms were obtained from the PlasmoDB database. Size of the bubble indicates the level of significance (1-p value) of the enriched GO term and colour density indicates the number of differentially expressed proteins (log2 protein count) associated with the GO term. (TIF) [file ppat.1012360.s009.tif]

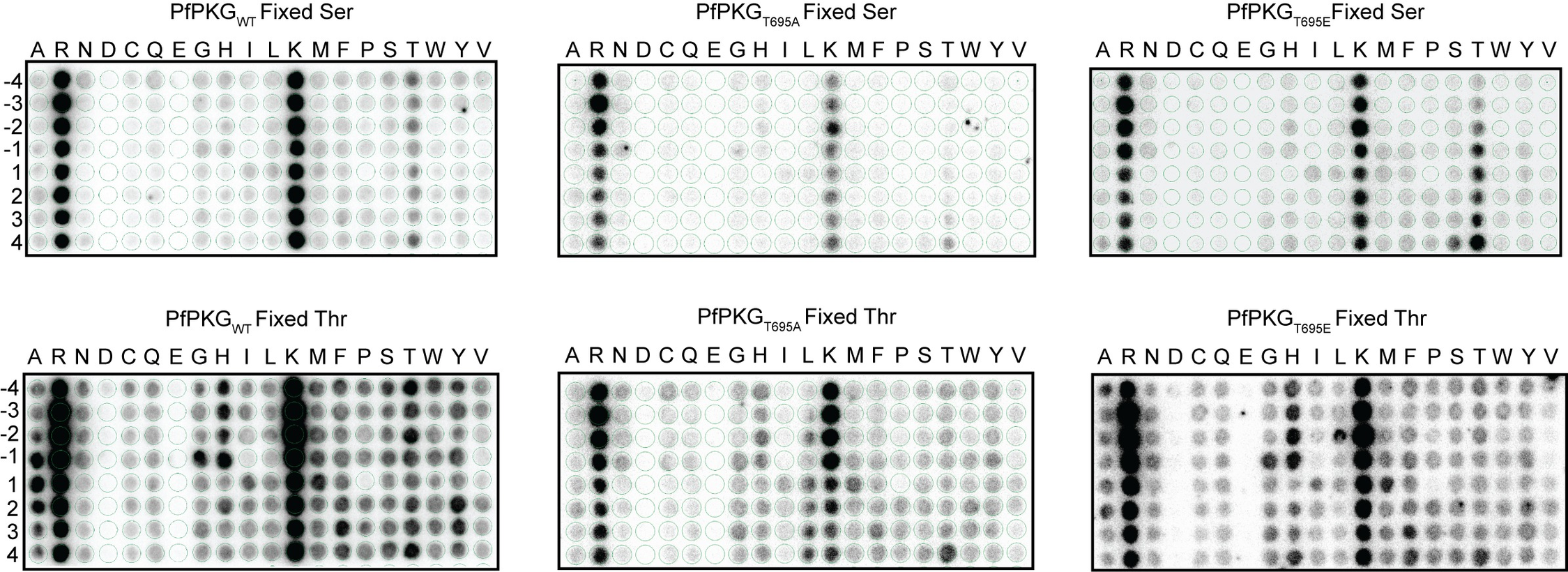

Supplement: S10 Fig — Autoradiographs of the oriented peptide array libraries showing phosphorylation activity of PfPKGWT, PfPKGT695A and PfPKGT695E. Library was based on the design A-X-X-X-X-S-X-X-X-X-A or A-X-X-X-X-T-X-X-X-X-A. (TIF) [file ppat.1012360.s010.tif]
