# Supplementary material for: Activation loop phosphorylation and cGMP saturation of PKG regulate egress of malaria parasites
Source: PLoS Pathog. 2024 Jun 27;20(6):e1012360. doi: 10.1371/journal.ppat.1012360 (PMC11236177; doi:10.1371/journal.ppat.1012360)
Supplement: S1 Table — (DOCX) [file ppat.1012360.s022.docx]

**Table S1**

| **Name** | **Sequence** |
| --- | --- |
| exon1_For | GAAGAAGATGATAATCTAAAAAAAGGG |
| Intron3_Rev | GTATGACCCTAAAAGGGGGG |
| exon4_Rev | gctctaaatgtacttctttgaactcc |
| wtpkg_For | CTGGTGAAACCATTGTTAAACAAGG |
| 5int_Rev | CTATTTACATGCATGTGCATGCAC |
| PKGsynth_For | CCGGTGGTGAACTGTATGACGC |
| 3int_Rev | GGTCATGTATGTTTAGAACCTGTAC |
| PKGutr_Rev | CCTTTCAATTATCATATCGCCC |
| St1_For | GGTTACGTGCGGTTAACAATAACTTCG |
| St1_Rev | GGACCCTGAATAATCTGCAGAAAAACG |
| T202A_For | GTGGTGAAGCCATTGTGAAAC |
| T202A_Rev | GTTTCACAATGGCTTCACCAC |
| Y214A_For | GATGTTCTGGCTATTCTGAAAGAG |
| Y214A_Rev | CTCTTTCAGAATAGCCAGAACATC |
| Y214F_For | GTGATGTTCTGTTCATTCTGAAAGAGGG |
| Y214F_Rev | CCCTCTTTCAGAATGAACAGAACATCAC |
| A214Y_For | GTGATGTTCTGTATATTCTGAAAGAGG |
| A214Y_Rev | CCTCTTTCAGAATATACAGAACATCAC |
| F96A_For | GCAGCTATTTCGCCATTATTAATAGCG |
| F96A_Rev | CGCTATTAATAATGGCGAAATAGCTGC |
| St4_For | GAACTGGTTACCGGTGGTGAAC |
| St4_Rev | CACAGACCTAGGAAAATCGATATCC |
| A694Y_For | GTCGTGCATATGCCCTGGTTGG |
| A694Y_Rev | CCAACCAGGGCATATGCACGAC |
| S576A_For | GTTAGCAAACGCGCCATTATCAATC |
| S576A_Rev | GATTGATAATGGCGCGTTTGCTAAC |
| St3_For | GCTCGAGCATGCATCTAGATTTTAT |
| gfp_Rev | CGTATGTTGCATCACCTTCAC |
| mCherry_Rev | GAACTCCTTGATGATGGCCATG |
| SH003 | AGCGGATCCCCCGGGATGGAAGAAGATGATAATCTA |
| SH004 | AGCCTCGAGTTAAAAATCTATGTCCCAGTTGTCTTC |
