# Supplementary material for: Activation loop phosphorylation and cGMP saturation of PKG regulate egress of malaria parasites
Source: PLoS Pathog. 2024 Jun 27;20(6):e1012360. doi: 10.1371/journal.ppat.1012360 (PMC11236177; doi:10.1371/journal.ppat.1012360)
Supplement: Attachment — Submitted filename: Response to Reviewers.docx [file ppat.1012360.s024.docx]

**Part I - Summary**
Please use this section to discuss strengths/weaknesses of study, novelty/significance, general execution and scholarship.

Reviewer #1: Koussis et al. describe the importance of phosphorylation of the seven residues essential within the CNB-B and the kinase domain of PKG and how they regulate PfPKG function. The authors used a conditional allelic replacement induced by rapamycin approach as well as recombinant expression of several PfPKG mutants and the wild type. The authors show that reduced cGMP binding to cyclic nucleotide-binding domain B does not affect in vitro kinase activity but does prevent parasite egress. Also, they identified T695 as an essential residue in the activation loop, contributing to PKG kinase regulation. However, the activation loop phosphorylation is not the mechanism for kinase activation by itself. Overall, this study provides important new insights into how PfPKG is regulated and in my opinion is an excellent fit for PLoS Pathogens.

Reviewer #2: cGMP signaling is a critical regulator of a number of processes in apicomplexan biology, including invasion, motility, and egress. While most well-studied model organisms have multiple cGMP effectors, parasites such as Plasmodium have a single effector kinase. In the present study, Koussis and coauthors dissect the an unusual regulation phosphorylation mechanism of PfPKG. There was much to like about this study, it is clearly written and logically presented. Overall I found the figures easy to parse and the data convincing.

Reviewer #3: This is a very thorough and systematic study of the role of phosphorylation sites in the cGMP-dependent kinase of the malaria parasite Plasmodium falciparum. The reverse genetics experiments are complex but well conducted and well-controlled. Some of the observations are surprising, but the data are solid. In particular, the dispensability of the phosphorylation of the activation T-loop residue T695 differs from the usual role of this phosphorylation in many other kinases (including AGCs). This means that PfPKG is regulated in a fundamentally divergent way to mammalian PKGs (but see comments below).

*We thank all three Reviewers for their positive comments regarding our manuscript.*

**Part II – Major Issues: Key Experiments Required for Acceptance**
Please use this section to detail the key new experiments or modifications of existing experiments that should be absolutely required to validate study conclusions.

Generally, there should be no more than 3 such required experiments or major modifications for a "Major Revision" recommendation. If more than 3 experiments are necessary to validate the study conclusions, then you are encouraged to recommend "Reject".

Reviewer #1: No major issues.

Reviewer #2: The authors appear to have missed an opportunity to combine their comparative changes in phosphoproteomics of their mutants with the in vitro OPAL peptide dataset. Do the subtle changes in the peptide dataset correlate with changes in the phosphoproteome (or are they at least mostly consistent?). If so, that would strengthen the paper. While it would be disappointing if that is not the case, it doesn't diminish the quality of the data, and would be important to explicitly note so alternative hypotheses can be considered by the field.

*The Reviewer raises a very interesting point here. We have examined possible correlations between the results of our phosphoproteome analysis and the OPAL datasets by using both the MEME analysis tool and the Phosphosite Plus database. Unfortunately we could not identify any significant correlation. However, disrupting PKG activity pharmacologically or genetically has a massive impact on the parasite phosphoproteome (PMID: 24594931, PMID: 33762339, PMID: 32359426 and PMID: 26149123) with many kinases acting downstream of PKG being impacted. Our failure to correlate the phosphoproteome data with the OPAL peptide data could be technical, with the abundance of phosphopeptides directly affected by our PKG mutations not being enriched to significant levels of detection. Furthermore, even if some peptides that correspond to the new consensus phosphorylation motifs were identified, it would be not be possible to prove in our study that these are direct substrates of PKG. In future studies,* *libraries of the specific peptides differentially phosphorylated in PKG mutants, could be screened to assess which ones can be directly phosphorylated by PKG and the respective mutants. Although this approach is still no proof of direct PKG phosphorylation in vivo, the results will form a comprehensive data set that could be further examined in vivo. To address this point, the following text has now been added in the Discussion (lines 522-527): “We were not able to correlate our phosphoproteome data with the OPAL substrate specificity data for enrichment of phosphopeptides with threonine in positions +1 to +4. This could be attributed to phosphopeptides directly phosphorylated by PKG not been enriched significantly.* *However our dataset paves the way for future experiments with libraries of differentially phosphorylated peptides identified from our PKG mutants.”*

Reviewer #3: MAJOR POINT:
Overall, this is a solid study with unexpected but well-supported outcomes. I have only one major suggestion, that relates to the aforementioned unexpected finding:

P. 18 and Fig 4H. The observation that the Tyr695Ala mutation does not affect kinase activity is surprising (in comparison to regulatory mechanisms in other AGC kinases where phosphorylation is a requisite for activity) but well supported by compelling data (Fig. 4H), Since there is an adjacent Tyrosine (Y694) or nearby Threonine (T699), phosphorylation of the latter may be required for activation. A double activation loop mutant (Y694-T695 or T695-T699, or a triple mutant), would be interesting to analyse.

*We fully agree with the reviewer that analysis of additional activation loop mutants could further delineate the importance of active site loop phosphorylation for PfPKG. Regarding Y694, the study of El Bakkouri et al (PMID:31239348) showed that mutation of Y694 to Alanine yielded very low levels of soluble protein, which was enzymatically inactive (PMID:31239348 - Supplementary Table 2). Based on the crystal structure, Y694 is involved in important interactions with K157 (K157–Y694 π-bond stabilization), perhaps explaining why its substitution in the El Bakkouri study led to inactive (likely misfolded) protein. In apparent conflict with that work, Y694 was identified as a phosphorylated residue in a global phosphoproteome study (PMID:22018241). However, since phosphorylation of Y694 should be detrimental to the K157-Y694 interaction, our view is that this was likely a misidentification, probably due to its proximity to T695. Collectively, it is for these reasons that we excluded analysis of Y694 from our recombinant analysis and from our in vivo studies. We do agree that future studies should focus on a detailed analysis of T699 in the activation loop both in vivo and in vitro (using single point phosphomutant and phosphomimetic mutants plus a double T695-T699 mutation), as suggested, since these data could provide further insights into the role of the activation loop in substrate specificity and PfPKG activation. However, since generation and analysis of transgenic P. falciparum parasites, in combination with recombinant expression of various mutants, requires many months of work, we respectfully suggest that these detailed studies should be a focus of future research.*

In other kinases of the AGC group, activation loop phosphorylation site ties together numerous regions of the protein through a hydrogen bonding network which includes the activation loop, the R125 form the catalytic loop, and the E/ F linker from the C-lobe, and a His in the C-helix from the N-lobe (see for example / summary Fig 1 of PMID: 22334660). It would be of interest to mention if the AGC-conserved residues that interact with the pT197 (in mammalian PKA) are conserved in PfPKG. If not, this would strongly support the notion that phosphorylation of the T in the activation loop has evolved to fulfill other functions than “conventional” enzyme activation. In this respect, the discussion of similarities with mammalian PKCδ (P. 25) is interesting. Are these “pT-interacting residues” conserved in PKCδ?

*This is an excellent suggestion; it emphasises a key finding of our work and is an issue that we should have addressed originally. To examine amino acid conservation in the activation loop of mammalian PKA and PfPKG, we overlayed the crystal structures of PfPKG (PDB: 5dyk) and mouse PKA (PDB:1atp). This identified significant differences between the two kinases that also extend to PKCδ. Accordingly, in our revised manuscript the following text has now been added to the Results section and an additional supplementary figure (Fig. S6) (lines 315-324) : “Active site loop phosphorylation coordinates defined kinase regions (C-helix, catalytic loop and activation loop) via a hydrogen bonding network [36, 37]. To examine if the activation loop of PfPKG shares structural similarities with well characterised members of the same family, the crystal structures of PfPKG (PDB: 5dyk) [19] and mouse PKA (PDB:1atp) [38] were overlayed (rmsd: 1.3Å) to look at the amino acid conservation within the activation loop phosphorylation site. As can be seen in Fig S6, two out of the five residues involved in the stabilization of the phosphate group via hydrogen bonding in PKA are not conserved in PfPKG. Residues H87 from the C-helix and T195 from the activation loop in PKA are replaced in PKG by N585 and A693 respectively.”.*

*Furthermore, the following text has been added in the Discussion (lines 507-515):* *“As we have shown (Fig. S6), the PKA residue network that stabilizes the phosphate group in the phosphorylation site is not fully conserved in PKG. Two out of the five residues involved in the stabilization of the phosphate group via hydrogen bonding in PKA are not conserved in PKG, since H87 and T195 in PKA, are replaced by N585 and A693 respectively. Interestingly these two residues (H87 and T195) are also not conserved in PKCδ and replaced by T394 and A505 respectively. These amino acid substitutions, particularly T195 (PKA) to A693 (PKG) or A505 (PKCδ) might contribute to a different mechanism underlying activation loop involvement in activation of these two protein kinases.”*

**Part III – Minor Issues: Editorial and Data Presentation Modifications**
Please use this section for editorial suggestions as well as relatively minor modifications of existing data that would enhance clarity.

Reviewer #1: I have only several minor revisions to request.
1. Please check the listing of phosphorylation residues in the text, sometimes the authors write T576 or S576.

*Phosphorylation residue 576 is now corrected as S576 throughout the manuscript*

2. In the Fourth paragraph of the introduction, please change from “two of these sites are found within CNB-B (T202, Y214) and four within the kinase domain” to “two of these sites are found within CNB-B (T202, Y214) and five within the kinase domain”.

*Corrected (line 93)*

3. Which concentration of Zaprinast was used? Please clarify that in the methods. Is this concentration toxic to the parasite?

*The zaprinast concentration used (75 μM) has now been added to the methods (lines 554-555). Zaprinast addition leads to premature egress which severely affects parasite invasion. In the context of the egress assays used in this work it was used solely as a tool to activate PKG as has been previously described in the literature (e.g. PMID: 23675297).*

4. Clarify why the authors extended the studies to F96.

*Previous studies using recombinant PfPKG (PMID: 12817987), have shown that mutations affecting cGMP binding to domains CNB-A and B have little or no effect on kinase activity. Our in vivo data with the Y214A substitution within CNB-B suggest that these small changes can have significant effects for the parasite in vivo, despite the fact that Y214 resides outside of the cGMP binding domain. Having identified this new mechanism, we wanted to extend our analysis to CNB-A. In CNB-A, the equivalent amino acid for Y214 is F96 and we wanted to test whether substitution of F96 with an alanine would have the same effect on parasites as the Y214A mutation. To clarify this reasoning in our revised manuscript, the revised version now reads (lines 172-174): “**To address the question of whether a similar mechanism exists for binding of cGMP to CNB-A, we extended our studies to CNB-A by substitution of F96, which occupies the position equivalent to Y214 in CNB-B.”.*

5. What is the cGMP saturation in PKG sites?

*As concentrations of cGMP increase, the CNB-D domain will become fully cGMP bound (saturated) long before the other domains, because it has a much higher affinity for cGMP. Studies by El Bakkouri et al* (*PMID:31239348), using isothermal titration calorimetry (ITC), showed binding affinities for recombinant CNB-A, -B, and -D domains of 1.4, 1.7 and 0.17 µM respectively. Similar studies by Kim et al (PMID: 25646845) using competition fluorescent polarisation measurements showed that the CNB-D domain binds cGMP with an EC50 of 38 nM, while A and B domains with an EC50 of 1 and 1.2 uM respectively.*

6. Please provide standard deviation (SD) data and apply statistics throughout. Data S1 needs to be improved with units and the number of replicates.

*We apologise for these omissions. Statistics have now been applied and Data S1 has been updated with units and number of replicates.*

7. The authors state that the binding of PKAtide substrate is not significantly different for WT PfPKG and PfPKGSTmut. It would be beneficial for the manuscript to provide statistical analysis supporting this conclusion.

*We are sorry for the omission. Statistical analysis is now provided to support our claims (Fig S4 and figure legends)*

Also, the authors state that despite their attempts, it was not possible to obtain reliable km values for ATP with PfPKGSTmut. Could the authors explain why?

*We believe that the main reason for our inability to obtain Km values for this mutant, is due to a constraint of the ADP-Glo Kinase assay, which can only be used at ATP concentrations up to 1 mM. The subtle mutations we have introduced, potentially modify the ATP binding pocket after cGMP activation in such a way that to obtain reliable values, the ATP concentration range would have had to be extended beyond 1 mM final concentration? to reliably determine the Km for PfPKGSTmut (under identical conditions using 50 μM PKAtide and 10 μM cGMP).*

8. In Figure 2H the authors state that there are differences between PfPKGWT and PfPKGSTmut in ML-10 and C2 inhibition values. However, no statistics are available to support the statement. The error bars are very high. Please review and revise as needed.

*We have revised and added statistical analysis to Fig. 2H.*

9. The authors need to improve the quality of Figures 3B and 3C. Figure S5F does not exist.

*Western blots are improved. We are sorry for the mistake regarding Figure S5. In the original version of the manuscript it should have mentioned “Figure S5C”. This is now corrected.*

10. Figures 3F and 3G do not match the textual description of the results. There are differences between DMSO- and RAP-treated parasites at 0.25 uM and 0.5 uM of C2. Please add statistics to corroborate the arguments.

*In the submitted version of our manuscript we mention “This showed that both RAP-treated lines were relatively insensitive to increased concentrations of C2 compared to the vehicle-treated lines”, which we feel matches Figure 3F and 3G as both RAP-treated lines grew in 0.25 μM C2 and line S576A_S817A also grew in 0.5 μM C2. DMSO-treated parasites (i.e WT) could not grow in these concentrations of C2, hence the negative fold change. However, we agree with the reviewer that our description was not clear. The following text has now been added in the revised manuscript (lines 263-268): “**Both RAP-treated lines were able to grow in the presence of 0.25 μM C2, while the RAP-treated pkgS817A/S819A_S817A line was also able to grow in 0.5 μM C2. By contrast the DMSO-treated lines (i.e. wild type), could not grow at these concentrations of C2, as expected.”*

*Parasitaemia values and the log fold transformations are now shown in Source Data with n=3. Statistical analysis is included in the new Source Data file but we have not included it in Figures 3F and 3G as we feel it would complicate the figure without providing any additional explanation. We would like to clarify that we are not comparing differences in growth but just showing that wild type parasites die in 0.25 and 0.5 μM C2, as PKG activity is inhibited, while RAP-treated parasites grow, which is evident from the negative and positive fold changes in parasitaemia. Additionally, both DMSO- and RAP-treated parasites grow in the presence of 0.125 μM C2 and vehicle (DMSO).*

11. Please add values units to all graphs. Also, it would be best to choose one standardized way to present the graph data in the Figures and Supplemental Figures.

*Throughout the manuscript we have kept all growth assays in the same format with the exception of the C2 experiments (Fig 3F, 3G) where we feel that standard growth curves would be very difficult to visualise. In our revised manuscript, all our kinetic assays now follow the same standardized values as requested. Values have been added to graphs and we have also changed the x-axis in our growth assays from “cycle” to “time” (h).*

12. Have the authors tested C2 and/or ML-10 against Y214A and Y214F mutant lines in order to check whether those mutations (situate away from the ATP binding site) can affect the potency of these compounds (even though the mutations are not in the ATP-binding pocket)? If so please mention.

*This is an interesting point raised by the reviewer. We have performed assays (n=4) with C2 and ML-10 against the Y214A mutant and found that this mutation does not affect the potency of the compounds. Our results are shown below:*

*Our data suggest that Y214A mutation in CNB-B does not affect (directly or indirectly) the ATP-binding pocket as would be expected. However we feel that it is not necessary to add these results to the revised manuscript as these assays were performed only to validate the quality of our kinetics data with this mutant recombinant protein. We hope that the reviewer agrees with us on this point.*

Reviewer #2: The structure models shown in Figure 1J are cluttered and difficult to interpret. As presented, the dots do not effectively show surfaces/packing, and the various colors of the cartoon portion are somewhat confusing. It is also important to differentiate between actual solved structure (the wild-type) and the two theoretical models that appear to have had the Y214 mutated but no additional modeling (e.g. energy minimisation, which is definitely unnecessary to make the author's point).

*The panel J in figure 1 is now redrawn for better readability and the PDB number has been added for the wild type PKG to differentiate between the actual crystal structure and our models. We hope the revised Figure 1J is easier to interpret. Please see also our response to Reviewer 3- point 2*.

Upon introduction of zaprinast it would be helpful to describe what the drug does for the non-specialist audience.

*We are sorry for not clarifying the mechanism of zaprinast. To clarify this, the revised version has the following sentence added (lines 163-164): “**Addition of zaprinast to schizonts induces egress by raising cGMP levels, leading to PKG activation and discharge of exonemes and micronemes.”*

Reviewer #3: In addition to that, the authors might want to consider the following (more minor) points:

P.11, top: “Replacement of Y694 with Ala likely weakens the packing of the kinase activation loop against the α-helix”. Say which alpha-helix, and what the consequences are.

*In our revised manuscript, we have added the following text to the manuscript to clarify the role of Y694 (lines 139-140): “**Replacement of Y694 with Ala likely weakens the packing of the kinase activation loop against the long α-helix (R149-D178) connecting the cyclic nucleotide-binding domain A (CNB-A) to CNB-B”. Regarding the consequences of this replacement, the following text has been added to the Discussion (lines 425-430) : “**The aliphatic chain of K157, which lies within the α-helix connecting CNB-A to CNB-B, performs a stacking interaction with the aromatic ring of Y694. The hydroxyl group of Y694 is engaged in hydrogen bonding with S160 Oγ. Substitution of Y694 with an alanine would remove these key stabilizing interactions and possibly release the PKG activation loop from the pentagonal frame, consequently affecting PKG function.”..*

P.11, middle: the fact the Tyr214->Phe substitution in CNB-B has no phenotype (while Tyr214->Ala is lethal) shows indeed that phosphorylation is not needed, but that the residue plays an important structural role. This is compounded by the fact that the corresponding position in CNB-A is a Phe. In Fig 1J, it is not clear how the Ala substitution affects the general environment (it is mentioned that “., and how it is different from the Phe substitution?... the only difference in the panels appears to be the substituted residue itself. Do the authors mean that stacking between F239 and Y214 (in the wild-type) stabilises the overall structure? If so, the mutation may cause lack of binding only due to general structural instability of the CNB. Please clarify (this is addressed in the discussion [P. 23], but could be clarified a bit more. CD analysis of this mutant (as has been done for PKGSTMUT) might be of interest to address the extent of destabilisation.

*We apologise for the lack of clarity regarding Y214A. The original text is replaced with new text (lines 453-460): “**Substitution of Y214 with Ala is predicted to disrupt the intramolecular stacking interactions present in CNB-B (Fig 1J). The phenyl ring of Y214 is mainly stabilised by pi-stacking interactions with F239 (parallel displaced) and by hydrophobic interactions involving the side chains of I203, V204 and V212. These core stabilizing interactions could still be maintained in the Y214F mutant due to the presence of its aromatic ring. By contrast, in the compact Y214A aliphatic mutant these interactions would be lost, leading to an intrinsic structural destabilisation of the CNB-B domain.”.*

*Regarding further biophysical analysis of the Y214A mutant, we do agree with the reviewer that it could further clarify the extent, if any significant, of destabilisation. However, our kinetic assays and inhibition assays with ML10 and C2 (please see our response to Reviewer 1, minor comment 12) suggest that maybe the overall fold of the kinase is minimally affected to be able to be detected by CD analysis.*

P.11: monitoring cleavage of SERA5 in quite an indirect proxy for PKG action… Could the PKAtide peptide used in subsequent experiments (Fig 1G) be used to monitor activity, if not in vivo, at least in least from parasites extracts? Also, why use SERA5 as a proxy here, and AMA1 in the experiments in Fig 4F? Please clarify.

*Although the PKAtide peptide can theoretically be used in experiments from parasite extracts there are technical difficulties that need to be taken into consideration. The main issue is that the same peptide can be phosphorylated by PKA since both these kinases share similar substrate specificities. This could potentially complicate analysis of the results. Regarding SERA5 processing, we fully agree that SERA5 processing and AMA1 relocalization are both indirect proxies of PKG activity. However, these assays measure two essential processes - exoneme discharge and microneme secretion - that are linked in vivo to PKG activation. We reasoned that in different mutants with complex phenotypes these assays could be used to dissect whether pathways known to be linked to PKG are affected. This is the reason that in our activation loop mutants we examined both SERA5 processing (Figure 4E) and AMA1 relocalization (Figure 4F).*

P. 15 and Fig 2G: “Attempts however to obtain reliable Km values for ATP with PfPKGSTmut, were unsuccessful, suggestive of subtle structural changes affecting ATP binding but not kinase activity in vitro”. It is mentioned in the Introduction that these sites can also bind cAMP. A wild idea: could it be that the mutations affect ATP binding properties of the CNBs?

*The mutations in our PKGSTmut are in CNB-B (T202) and in the kinase domain (S576, T695, S817 and S819). T202 is located outside of the cGMP binding site so theoretically it should not affect the binding properties of either cGMP or cAMP. Although the reviewer proposes a provocative hypothesis, we are not aware of ATP binding in the CNB domains of PfPKG. Structural studies of PvPKG with the nonhydrolyzable analog of ATP, adenylyl-imidodiphosphate (AMPPNP), did not show any binding of AMPPNP in any CNB. (although this could be due to the crystallography approach and associated technical limitations). What cannot be excluded and currently unknown are the global structural modifications to the intact PKG molecule upon cGMP binding. If these substantial modifications incurred can aid the binding of ATP in the CNBs, this is something that would be of interest in the kinase field and a future area of investigation.*

P. 18, bottom: the statement “These data suggest that T695 is phosphorylated well before the point of PKG activation at egress. It therefore appears unlikely that T695 phosphorylation contributes to the cGMP-mediated activation mechanism” is a bit misleading. Formally speaking, early phosphorylation might prime the enzyme for subsequent full activation mediated by cGMP binding at a later stage. In another words, this T-loop phosphorylation may be necessary, but not sufficient, for activation. The rest of the data show this is not the case, but this particular statement is logically flawed.

*We agree that the exact role, if any, of the early T695 phosphorylation is not addressed in our study and the second statement was premature. In the revised manuscript we have deleted the second phrase and it now reads (lines 34-342): “These data suggest that T695 is phosphorylated well before the point of PKG activation at egress.”*

P.20: “The increase in hyperphosphorylated sites observed in both T695A
and T695E phosphoproteomes raised the question of whether these mutations could
alter the substrate specificity of PfPKG”. Actually, the increase in hypophosphorylated sites raise the exact same question! :-) In the view of this reviewer, any change (increase/decrease/hypo/hyper) actually does.

*We fully agree that general deregulation of phosphosites could be due to the altered substrate specificity of PKG. Our purpose was to highlight the increase of hyperphosphorylated sites compared to the PKG knockout. However, our statement was not properly clarified. The revised manuscript now reads (line 383): “The differentially phosphorylated peptides…”*

Discussion: “The exact mechanism by which Y214A parasites overcome the lethal phenotype, in the presence of zaprinast, is not clear.” Could it be that the structural changes caused by the mutation have a direct effect (that does not require “release of the kinase domain”)?

*Like for PKG wild type, the PfPKGY214A mutant was successfully expressed in E.coli, indicating this mutation had a minimal impact on the protein overall fold. Nevertheless, discrete structural destabilisation in the CNB-B Y214A mutant likely decreased its binding affinity for cGMP. With zaprinast use, increased levels of cGMP due to phosphodiesterase inhibition, perhaps counteracted this effect by simply saturating the CNB-B binding pocket despite its lower affinity, hence restoring PKG function. It is also possible that the saturating levels of cGMP forced the CNB-B domain to a structural induced fit to bind cGMP optimally, hence also restoring PKG function. Activation of PfPKGY214A without release of the kinase domain it is a plausible and very interesting alternative. An active state PKG structure (ie with cGMP bound to it) would potentially provide an answer to this question as we would understand how the kinase domain is released in the proposed key-lock mechanism by El Bakkouri et al. Furthermore mutations could be modelled much more reliably to the active structure and see how changes in different CNBs could force alternative activation mechanisms.*

P.21, substrate preference: “We observed for both mutants a more relaxed specificity for Thr in positions -1 to +4” . Interestingly, the phosphopeptide corresponding to T695
(AYpTLVGTPHYMAPEVILGK) actually complies with that (T at +4). Could there be any significance to that?!

*This is an interesting observation that might warrant future examination. However, we would expect this particular residue to be hyperphosphorylated in both our mutants, while in contrast it was found to be significantly hypophosphorylated, following the same pattern as in our previously published PKG conditional knockout phosphoproteome (PMID:33762339)*

Discussion: “Future studies should explore whether activation loop phosphorylation is
an autophosphorylation event or whether it is mediated by PDK1”. Please mention which kinase is the Plasmodium PDK1 functional homologue.

*We apologise for this omission. The following text has been now added in the revised Discussion (lines 532-533) : “Future studies should explore whether activation loop phosphorylation is an autophosphorylation event or whether it is mediated by the P. falciparum orthologue of 3-phosphoinositide-dependent protein kinase-1 (PfPDK1 – Pf3D7_1121900)…”*

PLOS authors have the option to publish the peer review history of their article ([what does this mean?](https://journals.plos.org/plospathogens/s/editorial-and-peer-review-process#loc-peer-review-history)). If published, this will include your full peer review and any attached files.


If you choose “no”, your identity will remain anonymous but your review may still be made public.


**Do you want your identity to be public for this peer review?** For information about this choice, including consent withdrawal, please see our [Privacy Policy](https://www.plos.org/privacy-policy).

Reviewer #1: No

Reviewer #2: No

Reviewer #3: **Yes:** Christian Doerig
